# Increasing Colorectal Cancer Screening in Health Care Systems Using Evidence-Based Interventions

**DOI:** 10.5888/pcd15.180029

**Published:** 2018-08-09

**Authors:** Amy DeGroff, Krishna Sharma, Anamika Satsangi, Kristy Kenney, Djenaba Joseph, Katherine Ross, Steven Leadbetter, William Helsel, William Kammerer, Rick Firth, Tanner Rockwell, William Short, Florence Tangka, Faye Wong, Lisa Richardson

**Affiliations:** 1 Division of Cancer Prevention and Control, National Center for Chronic Disease Prevention and Health Promotion, Centers for Disease Control and Prevention, Atlanta, Georgia; 2 Diversified Business Consulting Group, Inc, Silver Spring, Maryland; 3 Information Management Services, Inc, Calverton, Maryland

## Introduction

Cancer is the second leading cause of death in the United States (1), and colorectal cancer (CRC) is the second leading cause of cancer death among cancers that affect both men and women (2). There is strong evidence that screening for CRC reduces incidence and mortality rates from the disease either by detecting cancer early, when treatments are more effective, or by preventing CRC through removal of precancerous polyps (3). The US Preventive Services Task Force recommends CRC screening for people at average risk (aged 50–75 y), using either stool-based tests (ie, fecal immunochemical test [FIT], fecal occult blood test [FOBT], multi-targeted stool DNA test [FIT-DNA]) or tests that directly visualize the colon (ie, colonoscopy, sigmoidoscopy, or computed tomographic colonography [CTC]) (3). Despite availability of these tests, a significant proportion of Americans remain unscreened; in 2016, only 67.3% of age-appropriate men and women were up to date with screening (4).

Although mortality rates from CRC have declined over time (5), disparities in incidence and mortality rates continue. In 2014, the most recent year for which data were available, the incidence of CRC among African Americans was 44.1 cases per 100,000, the highest rate among racial/ethnic groups (2). Similarly, the mortality rate of CRC among African Americans was 18.5 cases per 100,000, compared with 13.8 per 100,000 for whites (2). Disparities in incidence and mortality rates by socioeconomic factors, insurance status, and geographic areas are also well documented (6–8). With regard to CRC screening, disparities in screening persist with lower rates among people with low annual household income, with low educational attainment, and who are Hispanic/Latino (9). The National Colorectal Cancer Roundtable set an ambitious national target of 80% for CRC screening in the United States by 2018 (http://nccrt.org/).

**Figure 1:**
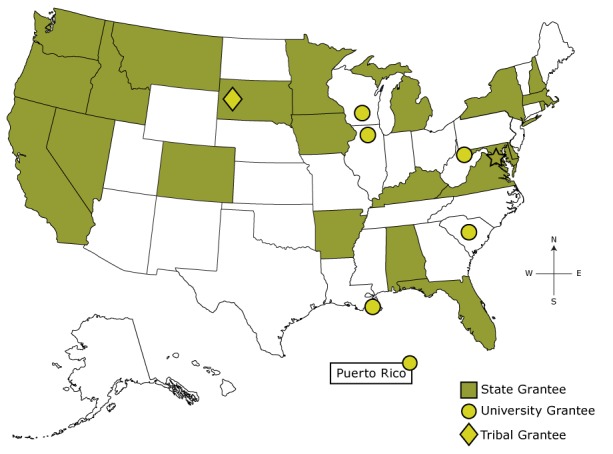
Map Showing Grantees of CDC’s Colorectal Cancer Control Program, Program Year 1, July 2015 through June 2016. Abbreviation: CDC, Centers for Disease Control and Prevention.

The Colorectal Cancer Control Program (CRCCP), funded by the Centers for Disease Control and Prevention (CDC), aims to increase CRC screening rates among medically underserved populations (www.cdc.gov/cancer/crccp/index.htm). The CRCCP funds 23 states, 6 universities, and 1 tribal organization (Figure 1) to partner with health care systems and implement evidence-based interventions (EBIs) recommended by the Community Preventive Services Task Force in the *Guide to Community Preventive Services* (Community Guide) (10). CDC is leading a comprehensive, multiple methods evaluation to address a range of process, outcome, and cost-related questions. In this article, we present evaluation results for the CRCCP’s first program year (PY1), July 2015 through June 2016. Data were collected from October 2015 through April 2017.

## Purpose and Objectives

CDC first funded the CRCCP from 2009 through 2015. In this earlier iteration, 22 states and 4 tribal grantees received funds to provide direct CRC screening services to low-income, uninsured, or underinsured populations known to have low CRC screening rates (11). Grantees contracted with primary care and gastroenterological providers to deliver recommended CRC screening tests. To a lesser degree, grantees implemented Community Guide–recommended EBIs with the goal of increasing population-level screening rates. Evaluation of this program focused on monitoring patient-level clinical service delivery, the types of EBIs implemented (12,13), costs (14,15), and changes in state-wide screening rates using data from the Behavioral Risk Factor Surveillance System (BRFSS). Evaluators found that program reach was insufficient to detect impact at the state level.

In response to the findings, CDC redesigned the CRCCP model and funded a new 5-year grant period beginning in 2015. Under the new model, grantees partner with primary care clinics to implement EBIs as well as supporting activities (SAs) such as health information technology (HIT) improvements to support population management for cancer screening. In contrast to the first CRCCP iteration in which the focus was primarily on individuals, changing to a health systems model increases public health impact because reach is extended (16). Grantees use public health data to identify and recruit primary care clinics serving low-income, high-need populations in their states. Under this new model, the clinic is the defined measurement unit, with clinic-level screening rates representing the primary outcome. CDC is conducting a comprehensive evaluation of the CRCCP to examine program processes, outcomes, and costs. The evaluation aims to support program improvement, strengthen accountability, and ensure sound policy decision making. In this article, we address 3 overarching evaluation questions:

- How many people are reached through the program?
- What EBI/SA activities are implemented by CRCCP grantees?
- Does the CRCCP contribute to improved screening rates in participating clinics?

## Intervention Approach

In 2010, CDC and the Health Resources and Services Administration (HRSA) commissioned the National Academy of Medicine to convene experts and examine the integration of public health and primary care (17). The premise of the study was that capacity in both public health and primary care could be expanded, and meaningful improvements in population health, including disparity reduction, could be achieved through effective integration. The resulting report identified CRC screening as an area for collaboration between public health and primary care, given the potential alignment in the goals of the CDC’s CRCCP and HRSA’s federally qualified health centers (FQHCs). CRCCP’s priority population is served by FQHCs, and CRC screening rates in these clinics are often low. The national CRC screening rate in 2016 for FQHCs was 39.9% (18). In addition, HRSA recognized the importance of CRC screening and had recently introduced a new quality measure for CRC screening that FQHCs were required to report annually. These circumstances offered the opportunity for FQHCs and local public health agencies to collaborate and achieve greater increases in screening.

Along with public health and primary care integration, several tenets of effective public health implementation also support the CRCCP model (19). These include focusing on defined, high-need populations in which disease burden is highest; establishing partnerships to support implementation; implementing sustainable health system changes; using evidence-based strategies to maximize scarce public health resources; encouraging innovation in adaptation of EBIs/SAs; conducting ongoing, systematic monitoring and evaluation; and using data for program improvement and performance management.

**Figure 2:**
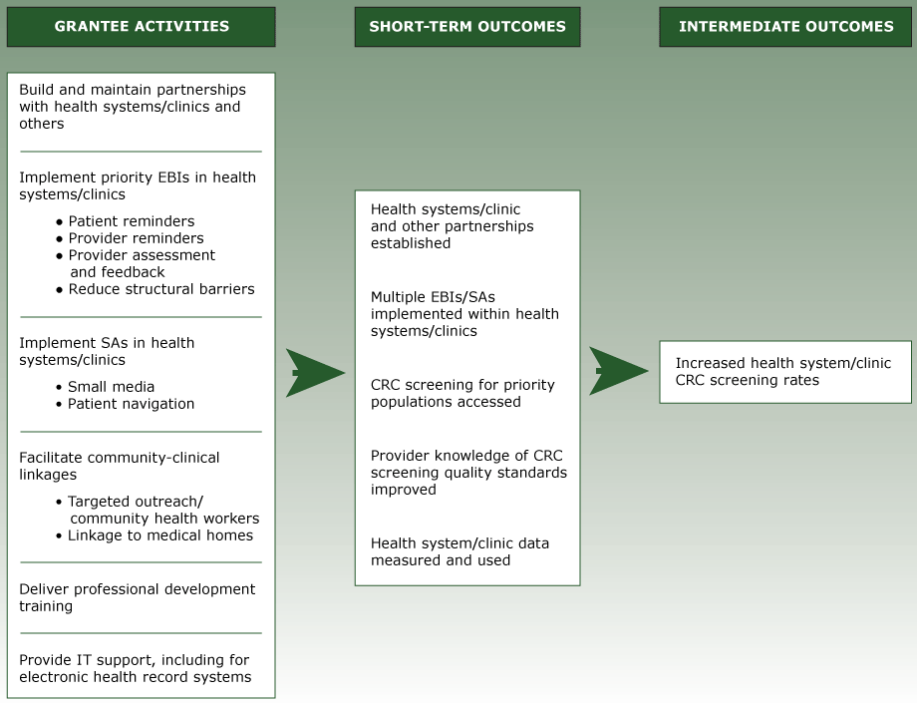
Program Logic Model Showing Activities and Outcomes of the Colorectal Cancer Control Program, Program Year 1, Centers for Disease Control and Prevention, July 2015 through June 2016. Abbreviations: CRC, colorectal cancer; EBIs, evidence-based interventions; SAs, supporting activities.

The program logic model (Figure 2) reflects the activities, outputs, and short-term outcomes for the CRCCP. Along with health system clinics, grantees partner with organizations in their states such as primary care associations, the American Cancer Society, and organizations that can assist with implementation, evaluation, or both. Grantees are required to implement 2 or more EBIs identified in the Community Guide in each clinic (Table 1). CDC prioritizes 4 EBIs including patient reminders, provider reminders, provider assessment and feedback, and reducing structural barriers. Two SAs (ie, small media, patient navigation) can be implemented alongside the priority EBIs, and grantees are encouraged to conduct provider education and community outreach to link priority population members to clinical services. Grantees use HIT to integrate EBIs at the systems level (eg, provider receives an automated reminder via the electronic health record [EHR] while seeing a patient) and address issues that interfere with accurate screening rate measurement (eg, entering screening information in incorrect EHR fields) (20).

**Table 1:** Evidence-Based Interventions and Supporting Activities Used by Grantees, Program Year 1, CDC Colorectal Cancer Control Program, July 2015–June 2016

[CATEGORY NAME]	Definition^a^
**Evidence-Based Interventions**	
Patient reminders	Patient reminders or recalls are text-based (ie, letter, postcard, e-mail) or telephone messages advising people that they are due (reminder) or overdue (recall) for screening. Reminder messages may be general to address an overall priority population or tailored to specific individuals.
Provider reminders	Reminders inform health care providers it is time for a patient’s cancer screening test (reminder) or that the patient is overdue for screening (recall). The reminders can be provided in different ways, such as patient charts or by e-mail.
Provider assessment and feedback	Provider assessment and feedback interventions both evaluate provider performance in offering and/or delivering screening to patients (assessment) and present providers with information about their performance in providing screening services (feedback). Feedback may describe the performance of a group of providers or an individual provider and may be compared with a goal or standard.
Reducing structural barriers	Structural barriers are noneconomic burdens or obstacles that impede access to screening. Interventions designed to reduce these barriers may facilitate access to cancer screening services by reducing time or distance between service delivery settings and target populations, modifying hours of service to meet patient needs, offering services in alternative or nonclinical settings, or eliminating or simplifying administrative procedures and other obstacles.
**Supporting Activities**	
Small media	Small media include videos and printed materials such as letters, brochures, and newsletters. These materials can be used to inform and motivate people to be screened for cancer. They can provide information tailored to specific individuals or targeted to general audiences.
Patient navigation	Patient navigation is a strategy aimed at reducing disparities by helping patients overcome barriers to health care. For purposes of the CRCCP, patient navigation is defined as individualized assistance offered to patients to help overcome health care system barriers and facilitate timely access to quality screening and follow-up, as well as initiation of treatment services for people diagnosed with cancer. Patient navigation includes assessment of patient barriers, patient education, resolution of barriers, and patient tracking and follow-up. Patient navigators may be professional (eg, nurse) or lay workers.
Professional development/provider education	Professional development/provider education are interventions directed at health care staff and providers to increase their knowledge and to change attitudes and practices in addressing cancer screening. Activities may include distribution of provider education materials, including screening recommendations, and/or continuing medical education opportunities.
Community health workers	Community health workers are lay health educators with a deep understanding of the community and are often from the community being served. Community health workers work in community settings in collaboration with a health promotion program, clinic, or hospital to educate people about cancer screening, promote cancer screening, and provide peer support to people referred to cancer screening.

Abbreviations: CDC, Centers for Disease Control and Prevention; CRCCP, Colorectal Cancer Control Program.

a Based on definitions from *The Guide to Community Preventive Services*.

## Evaluation Methods

Using CDC’s Framework for Program Evaluation (21), we developed a comprehensive evaluation to assess processes and outcomes for the 5-year program period. The 6-step framework includes 1) engaging stakeholders, 2) describing the program, 3) focusing the evaluation design, 4) gathering credible evidence, 5) justifying conclusions, and 6) ensuring use and sharing lessons learned. Stakeholders, including CRCCP grantees, CDC staff, and health care experts, provided guidance throughout the evaluation planning process. The program logic model helped to describe the program and focus the evaluation design. In developing the evaluation plan, evaluators specified key questions and selected appropriate methods to address them. The multiple methods evaluation includes an annual grantee survey (Office of Management and Budget [OMB] control no. 0920–1074), a clinic-level data set (OMB control no. 0920–1074), case studies, cost studies, and use of secondary data (eg, financial reports). The description of methods centers on the collection, reporting, and analysis of clinic-level data presented in this article.

For the clinic data set, we developed a detailed data dictionary including record identification numbers, health system and clinic characteristics, patient population characteristics, screening rate measures, monitoring and quality improvement activities, and EBIs/SAs. Five grantees reviewed and provided feedback on the data dictionary. To support consistent and accurate reporting of clinic-level CRC screening rates, we developed *Guide for Measuring Cancer Screening Rates in Health Systems Clinics* (www.cdc.gov/cancer/crccp/guidance_measuring_crc_screening_rates.htm). The guide provides information for calculating and validating CRC screening rates using chart review–generated or EHR-generated rates. Grantees use 1 of the following 4 nationally recognized screening rate measures: 1) National Committee for Quality Assurance’s Healthcare Effectiveness Data and Information Set (HEDIS) (www.ncqa.org/hedis-quality-measurement), 2) HRSA’s Uniform Data System (UDS) (https://bphc.hrsa.gov/datareporting/), 3) Indian Health Service’s Government Performance and Results Act (www.ihs.gov/crs/gprareporting/), or 4) the National Quality Forum’s endorsed measure (www.qualityforum.org/Measures_Reports_Tools.aspx). Each measure has specifications for the numerator and denominator used to calculate the screening rate. The 4 options are provided to accommodate varying reporting requirements of grantees’ clinic partners (eg, FQHCs must report UDS screening rates to HRSA). For any given clinic, grantees must specify at baseline their selected CRC screening rate measure and 12-month measurement period (eg, calendar year). This same screening rate measure and measurement period must be used consistently for annual reporting. We encourage grantees to validate EHR-calculated screening rates using the chart review methods outlined in the guidance and, when appropriate, to partner with HIT experts to improve EHR data systems for monitoring and reporting CRC screening rates.

Baseline data are collected at the time a clinic is recruited for CRCCP participation. Annual data are reported each September following the end of the program year (July–June). This reporting provides CDC a longitudinal data set to examine EBI/SA implementation over time and assess changes in CRC screening rates. We developed spreadsheet-based forms, one each for clinic baseline and annual data. Grantees may use these forms to collect data directly from clinics or send the forms to clinic staff to complete and return. The forms incorporate validation features such as specified data ranges and drop-down response boxes (eg, primary CRC screening test type). The data collection tools were pilot-tested with 5 grantees for clarity, feasibility, and functionality.

Grantees use a web-based data reporting system, Clinic Baseline and Annual Reporting Systems (CBARS), developed by CDC’s data contractor, Information Management Services (IMS), to report clinic data to CDC. CBARS has built-in features to improve data quality including identifying missing data fields, flagging errors, and assessing discrepancies between historical and current responses. Data fields (eg, changes in clinic population size) can be updated at any time. Grantees were trained on the data variables, forms, and CBARS through CDC-led webinars. We provide on-going technical assistance to grantees and maintain a summary of frequently asked questions for use by grantees.

The clinic data can be divided into 3 categories: clinic characteristics, process implementation, and CRC screening rates. Clinic characteristics include clinic type, clinic size based on screening-eligible (ages 50–75 y) patient count, percentage of uninsured patients, primary CRC screening test type used by the clinic, availability of free fecal testing kits for patients, patient-centered medical home recognition, and rurality based on the US Department of Agriculture’s rural–urban continuum codes (22).

Process implementation variables include several related to EBI and SA activities. At baseline, grantees report whether each EBI/SA is in place before CRCCP implementation, regardless of the quality, reach, or level of functionality. Annually, grantees report whether the EBI/SA is in place at end of program year and whether CRCCP resources were used during the program year toward the EBI/SA. We define CRCCP resources as funds, staff time, materials, or contracts used to contribute to planning, developing, implementing, monitoring, evaluating, or improving an EBI/SA. If an EBI/SA was reported as not in place at the end of the program year, grantees report whether planning activities to implement the EBI/SA in the future were conducted. Analyzing these data allows CDC to assess whether CRCCP resources were used to implement a new EBI/SA in the program year (ie, EBI/SA was not in place at baseline), enhance an existing EBI/SA (ie, the EBI was in place at baseline and CRCCP resources were used to improve the EBI’s implementation during the program year), or plan for future implementation of the EBI/SA.

Other process implementation variables include the existence of a CRC screening policy and CRC clinic champion. A champion is an individual who takes a leadership role in a public health effort. Other variables include frequency of monitoring the CRC screening rate and frequency of implementation support provided to the clinic. Implementation support is defined as onsite or other (eg, telephone) contacts with the clinic to support and improve implementation activities for EBIs/SAs and CRC screening data quality.

The third category, CRC screening rates, includes the 12-month measurement period, screening rate measure used, numerator and denominator to calculate the screening rate, and if chart review is used, the percentage of charts extracted. Grantees also report a screening rate target for the upcoming program year.

We used descriptive analyses to summarize clinic characteristics and process implementation. We calculated a weighted average of baseline and annual screening rates across clinics, where weights were the clinic screen-eligible patient counts, the screening rate denominators reported at baseline and again at the end of PY1. Screening rate change was calculated as the difference between the weighted baseline screening rate and weighted PY1 screening rate. We calculated the number of patients screened at each clinic by multiplying the clinic screening rate by the respective screen-eligible patient count. Weighted screening rates and screened patient counts were determined by clinic characteristics (eg, rurality, size) and by process implementation status (eg, number of EBIs supported by CRCCP resources). All data analyses were conducted using SAS software, version 9.3 (SAS Institute Inc).

## Results

In PY1, 29 of the 30 CRCCP grantees reported data for at least 1 clinic; 1 grantee did not recruit any clinics in PY1. A baseline and annual record was reported for each of 418 clinics. We excluded 5 clinics because grantees had terminated the partnership before the end of PY1, leaving a total of 413 clinics for analysis. Grantees reported baseline and PY1 annual screening rate data for 387 of the 413 (93.7%) clinics.

The 413 clinics represent 3,438 providers serving a CRC screening-eligible population of 722,925 patients. The recruited clinics represent 140 unique health systems. Of the 413 clinics, most were FQHCs or Community Health Centers (CHCs) (71.9%); certified patient centered medical homes (73.1%); and located in metro areas (72.4%). The clinics varied in size, with 27.4% of clinics serving fewer than 500 patients; 36.8% serving between 500 and 1,500 patients; and 35.8% serving more than 1,500 patients (Table 2). The proportion of uninsured patients within clinics also varied; 30.8% of clinics reported large uninsured patient populations (more than 20%). More than half (52.5%) used FIT/FOBT as their primary CRC screening test, and 28.8% had access to free fecal test kits. At baseline, many clinics had at least one EBI (87.9%) or SA (72.6%) already in place.

**Table 2:** Characteristics of Participating Primary Care Clinics (N = 413), Program Year 1, CDC Colorectal Cancer Control Program, July 2015–June 2016

Clinic Characteristic	Percentage of Clinics^a^ (No.)
**Clinic type**	
Community health center/federally qualified health center	71.9 (297)
Health system/hospital owned	15.7 (65)
Private/physician owned	6.1 (25)
Other primary care facility	6.3 (26)
**Patient-centered medical home recognized**	
Yes	73.1 (302)
No	24.7 (102)
Unknown	2.2 (9)
**Rurality^b^**	
Metro	72.4 (299)
Urban	20.1 (83)
Rural	5.8 (24)
Unknown	1.7 (7)
**Clinic size (no. of patients)^c^**	
Small (<500)	27.4 (113)
Medium (500–1,500)	36.8 (152)
Large (>1,500)	35.8 (148)
**Uninsured patient population status (%)**	
Low (<5)	35.4 (146)
Medium (5–20)	28.1 (116)
High (>20)	30.8 (127)
Unknown	5.8 (24)
**Primary colorectal cancer test type**	
FIT/FOBT	52.5 (217)
Colonoscopy referral	32.2 (133)
Varies by provider	12.3 (51)
Unknown	2.9 (12)
**Free fecal testing kits**	
Yes	28.8 (119)
No	64.7 (267)
Unknown	6.5 (27)
**Number of evidence-based interventions in place at baseline**	
0	12.1 (50)
1	20.1 (83)
2	16.9 (70)
3	30.3 (125)
4	20.6 (85)
**Number of supporting activities in place at baseline**	
0	27.4 (113)
1	27.8 (115)
2	22.8 (94)
3	21.8 (90)
4	0.2 (1)

Abbreviations: CDC, Centers for Disease Control and Prevention; FIT/FOBT, fecal immunochemical test/fecal occult blood test.

a Percentages are unweighted and may not sum to 100% because of rounding.

b Based on US Department of Agriculture’s rural–urban continuum codes.

c Based on count of eligible patients aged 50 to 75 years.

During PY1, grantees used CRCCP resources to implement new or to enhance EBIs in 95.2% of clinics. Patient reminder activities were supported most frequently (73.1%), followed by provider assessment and feedback (64.9%), reducing structural barriers (53.0%), and provider reminders (47.7%) (Table 3). All 4 EBIs were more often enhanced than implemented as a new activity. CRCCP resources were used less often to plan future EBI activities.

**Table 3:** Status of Process Implementation (Evidence-Based Interventions and Supporting Activities) Performed by Primary Care Clinics (N = 413), Program Year 1^a^, CDC Colorectal Cancer Control Program, July 2015–June 2016

Activity	Clinics Using CRCCP Resources^b^	Implemented New Activity	Enhanced Existing Activity	Planning-Only Activity	Unknown
	% (No.)				
**Evidence-based interventions**	95.2 (393)	—	—	—	—
Patient reminders	73.1 (302)	29.5 (89)	54.3 (164)	12.9 (39)	3.3 (10)
Provider reminders	47.7 (197)	20.3 (40)	64.0 (126)	8.6 (17)	7.1 (14)
Provider assessment and feedback	64.9 (268)	30.2 (81)	51.9 (139)	10.8 (29)	7.1 (19)
Reducing structural barriers	53.0 (219)	29.2 (64)	38.8 (85)	30.1 (66)	1.8 (4)
**Supporting activities**	86.4 (357)	—	—	—	—
Provider education	57.6 (238)	35.7 (85)	42.4 (101)	19.8 (47)	2.1 (5)
Small media	69.0 (285)	43.2 (123)	42.8 (122)	10.2 (29)	3.9 (11)
Community health workers	11.6 (48)	27.1 (13)	25.0 (12)	47.9 (23)	0
Patient navigators	43.8 (181)	19.3 (35)	31.5 (57)	48.6 (88)	0.6 (1)

Abbreviations: CDC, Centers for Disease Control and Prevention; CRCCP, Colorectal Cancer Control Program.

a Percentage estimates are unweighted and may not sum to 100% because of rounding.

b Clinics could use CRCCP resources to implement, enhance or plan for the chosen activity.

CRCCP resources were used toward SAs in 86.4% of clinics. Resources were used to support small media most frequently (69.0%), followed by provider education (57.6%) (Table 3). Only 11.6% of clinics used resources for supporting community health workers. However, nearly half of the clinics conducted planning activities for future implementation of community health workers (47.9%) and patient navigators (48.6%). Provider education was more often enhanced than newly implemented (42.4% vs 35.7%), as were patient navigators (31.5% vs 19.3%).

Most clinics reported having a CRC screening champion (78.7%) and a CRC screening policy (72.6%) in place at the end of PY1 (Table 4). Most clinics received implementation support from the CRCCP grantees on a weekly (12.3%) or monthly (77.7%) basis. Clinics monitored CRC screening rates at different intervals, including monthly (63.4%) or quarterly/semi-annually/annually (34.5%). Most clinics (73.1%) performed screening rate validation using chart review or other methods as part of CRCCP implementation.

**Table 4:** Other Program Implementation Factors in Participating Clinics (N = 413), Program Year 1, CDC Colorectal Cancer Control Program, July 2015–June 2016

Other Program Element	Percentage of Clinics^a^ (No.)
**Colorectal cancer screening champion**	
Yes	78.7 (325)
No	18.9 (78)
Unknown	2.4 (10)
**Colorectal cancer screening policy**	
Yes	72.6 (300)
No	25.7 (106)
Unknown	1.7 (7)
**Frequency of implementation support**	
Weekly	12.3 (51)
Monthly	77.7 (321)
Quarterly, semi-annually, or annually	9.9 (41)
**Frequency of screening rate monitoring**	
Monthly	63.4 (262)
Quarterly, semi-annually, or annually	34.5 (151)
**Performs screening rate validation**	
Yes	73.1 (302)
No	18.9 (78)
Unknown	8.0 (33)

Abbreviation: CDC, Centers for Disease Control and Prevention.

a Percentage estimates are unweighted; do not necessarily sum to 100% because of rounding.

Table 5 provides screening rates overall and by key clinic characteristics at baseline and PY1, as well as screening rate changes from baseline to PY1 for the 387 clinics reporting baseline and PY1 screening rates. A total of 640,086 patients were eligible for screening at baseline, and 631,634 patients were eligible at the end of PY1. The average screening rate increased during PY1 by 4.4 percentage points from baseline (42.9%) to PY1 (47.3%). The total number of patients up to date with CRC screening was 274,694 at baseline and 298,790 at the end of PY1, an increase of 24,096 patients, which represents 3.8% of the baseline eligible patient counts.

**Table 5:** Colorectal Cancer Screening–Eligible Patient Population Counts and Weighted Screening Counts, Changes From Baseline to Program Year 1^a^ (N = 387), CDC Colorectal Cancer Control Program, July 2015–June 2016

Characteristic	No. of Clinics	Baseline Screening–Eligible Patient Counts	PY1 Screening-Eligible Patient Counts	Baseline SR^b^ (%)	Baseline Screened Patient Counts	PY1 SR^b^ (%)	PY1 Screened Patient Counts	Change^c^ in SR	Change in Screened Patient Counts^d^ (%^e^)
**Overall**	387	640,086	631,634	42.9	274,694	47.3	298,790	4.4	24,096 (3.8)
**Clinic type**									
FQHC/CHC	284	373,405	372,878	36.5	136,469	41.9	156,417	5.4	19,948 (5.3)
Health system/hospital	58	180,498	176,541	58.9	106,368	61.5	108,554	2.6	2,186 (1.2)
Private/physician owned	22	48,868	44,416	42.3	20,688	41.5	18,417	−0.8	−2,271 (−4.6)
Other primary care facility	23	37,315	37,799	29.9	11,170	40.7	15,402	10.8	4,232 (11.3)
**Rurality** f									
Metro	280	493,124	491,916	43.8	216,209	47.7	234,610	3.9	18,400 (3.7)
Urban	77	112,765	107,890	41.9	47,256	47.8	51,586	5.9	4,330 (3.8)
Rural	23	21,833	18,529	38.3	8,363	50.3	9,313	12.0	949 (4.3)
Unknown	7	12,363	13,300	23.2	2,865	24.7	3,281	1.5	416 (3.4)
**Clinic size (no. of patients)**									
Small (<500)	103	31,108	35,387	28.0	8,701	29.2	10,328	1.2	1,627 (5.2)
Medium (500–1,500)	142	125,523	126,694	32.7	40,990	40.4	51,179	7.7	10,189 (8.1)
Large (>1,500)	142	483,455	469,553	46.5	225,003	50.5	237,283	4.0	12,280 (2.5)
**Uninsured patient population status (%)**									
Low (<5)	140	305,362	303,681	48.4	147,748	51.5	156,460	3.1	8,712 (2.9)
Medium (5–20)	113	165,359	160,929	39.1	64,664	46.0	74,072	6.9	9,408 (5.7)
High (>20)	113	139,007	143,942	38.7	53,825	41.4	59,556	2.7	5,731 (4.1)
Unknown	21	30,358	23,082	27.9	8,457	37.7	8,702	9.8	245 (0.8)
**Primary CRC test type**									
FIT/FOBT	212	249,597	249,057	32.7	81,634	39.0	97,028	6.3	15,395 (6.2)
Colonoscopy	118	317,712	311,704	52.4	166,565	55.1	171,617	2.7	5,053 (1.6)
Varies by provider	47	60,829	51,697	39.1	23,765	43.6	22,529	4.5	−1,236 (−2.0)
Unknown	10	11,947	19,177	22.9	2,730	39.7	7,615	16.8	4,885 (40.9)
**Free fecal testing kits**									
Yes	117	176,019	167,969	35.5	62,563	42.2	70,800	6.7	8,237 (4.7)
No	247	411,856	415,706	44.7	184,044	48.3	200,812	3.6	16,768 (4.1)
Unknown	23	52,211	47,959	53.8	28,087	56.7	27,178	2.9	−909 (−1.7)
**Number EBIs supported with CRCCP during PY1**									
0	19	30,249	31,748	48.4	14630	48.6	15,434	0.2	805 (2.7)
1	109	230,943	233,202	50.6	116898	52.1	121,432	1.5	4,533 (2.0)
2	66	113,239	113,127	38.8	43943	43.1	48,779	4.3	4,836 (4.3)
3	82	95,580	99,989	42.4	40549	50.4	50,363	8.0	9,814 (10.3)
4	111	170,075	153,569	34.5	58674	40.9	62,782	6.4	4,108 (2.4)
**CRC screening champion**									
Yes	301	523,200	521,724	43.1	225,517	48.0	250,475	4.9	24,957 (4.8)
No	76	95,419	89,567	39.8	38,011	40.5	36,270	0.7	−1,742 (−1.8)
Unknown	10	21,467	20,344	52.0	11,166	59.2	12,046	7.2	880 (4.1)
**CRC screening policy**									
Yes	294	456,376	447,686	42.2	192,603	47.7	213,766	5.5	21,163 (4.6)
No	89	181,604	181,350	45.1	81,913	46.6	84,553	1.5	2,640 (1.5)
Unknown	4	2,105	2,598	8.5	179	18.1	471	9.6	292 (13.9)

Abbreviations: CDC, Centers for Disease Control and Prevention; CRC, colorectal cancer; CRCCP, Colorectal Cancer Control Program; EBIs, evidence-based interventions; FIT/FOBT, fecal immunochemical test/fecal occult blood test; FQHC/CHC, federally qualified health center/community health center; PY1, program year 1.

a Restricted to clinics that provided both baseline and PY1 screening rates.

b Screening rate averages were weighted by screening eligible patient counts.

c Change was calculated as the percentage point difference between baseline screening rate and PY1 screening rate.

d Change was calculated as the difference between PY1 screened patient counts and baseline screened patient counts.

e Change in number of patients from baseline to PY1 as percentage of baseline eligible patient counts.

f Based on US Department of Agriculture’s rural–urban continuum codes.

Baseline screening rates varied by clinic type. Health system/hospital clinics had a higher baseline screening rate (58.9%) than FQHCs/CHCs (36.5%), private/physician owned clinics (42.3%) or other primary care facilities (29.9%). During PY1, FQHCs/CHCs and other primary care facilities observed a larger increase in screening rates (5.4 and 10.8 percentage points, respectively), than health system/hospital clinics and private/physician owned clinics (2.6 and −0.8 percentage points, respectively).

Although rural clinics had the lowest average baseline screening rate at 38.3%, their screening rate during PY1 increased by 12.0 percentage points, higher than those of metro or urban clinics. The baseline screening rate was highest among large clinics (46.5%), followed by medium clinics (32.7%) and small clinics (28.0%). The average screening rate increase during PY1 was greatest among medium-sized clinics (7.7 percentage points) compared small and large clinics (1.2 and 4.0 percentage points, respectively).

Baseline screening rates and screening rate increases also varied by the proportion of clinic patients that were uninsured. Among clinics reporting their uninsured patient population, the baseline screening rate was lowest (38.7%) among clinics with a high uninsured patient population (more than 20%). However, clinics with 5% to 20% uninsured patients had the largest percent increase in screening (6.9 percentage points) during PY1. Among clinics reporting primary screening test type, clinics using FIT/FOBT observed greater screening rate increases (6.3 percentage points) than those clinics primarily using colonoscopy (2.7 percentage points). Clinics that reported having free fecal testing kits available for patients observed greater screening rate increases than those without (6.7 vs 3.6 percentage points).

Although PY1 screening rates varied by the number of EBIs newly implemented or enhanced in PY1, the highest screening rate increases were observed among clinics newly implementing or enhancing 3 or 4 EBIs (8.0 and 6.4 percentage points, respectively). Among clinics reporting their status of CRC screening champion or CRC screening policy in place at the end of PY1, clinics with a champion or screening policy reported greater increases in screening rates (4.9 and 5.5 percentage points, respectively) than clinics without them (0.7 and 1.5 percentage points, respectively).

## Implications for Public Health

With the goal of increasing CRC screening and reducing disparities, the CRCCP integrates public health and primary care, implementing evidence-based strategies in clinics to achieve sustainable health systems change. Early results from our PY1 evaluation, including changes in screening rates, suggest the CRCCP is working; program reach was measurable and substantial, clinics enhanced EBIs in place or implemented new ones in clinics, and we observed an increase in the overall average screening rate.

Our data suggest that the CRCCP is reaching its intended population. At baseline, the screening rate was low, at only 42.9%, and nearly three-quarters of the 413 clinics were FQHCs/CHCs. Of interest, 92.5% of clinics were located in metro or urban areas. Baseline screening rates were lowest in rural clinics, and evidence indicates that death rates for CRC are highest among people living in rural, nonmetropolitan areas (23); therefore, expansion of the program to rural areas is important. The diversity observed in other clinic characteristics such as clinic size (patients aged 50–75 y) and percentage of uninsured patients was expected, given the varied and unique contexts in which grantees are operating. Reach will continue to expand as additional clinics participate in years 2 through 5.

Consistent with the new model, grantees committed CRCCP resources during PY1 toward EBI implementation in 95% of all participating clinics. However, less than 50% of clinics used CRCCP resources for provider reminders in PY1. Provider reminders can increase screening rates by a median of 15.3% (24). If reminders are integrated into an electronic health system, the activity is sustainable. Consequently, grantees could prioritize provider reminders for clinics where implementation is poor or not yet instituted.

Among the 387 clinics for which screening rate changes were calculated, 50.0% had either 3 or 4 EBIs in place at the end of the first program year. Using multiple EBIs that combine different approaches to increase community demand and access to cancer screening leads to greater effects (25). Grantees could be encouraged to newly implement or improve EBIs consistent with this finding. Interestingly, large numbers of clinics had EBIs in place at baseline, therefore, grantees more often expended CRCCP resources to enhance implementation of existing EBIs than establish new ones. That resources were used toward these existing EBIs suggests the potential importance of public health intervention to improve and scale up implementation of these activities. A case study is under way that will help us understand the ways in which EBIs are enhanced.

Grantees complemented EBI implementation with extensive SAs; CRCCP resources were used for SAs in more than 80% of clinics. Small media, which was used most often, can be distributed with patient reminders by community health workers and patient navigators to strengthen those strategies. Among the 181 clinics where CRCCP resources were used toward patient navigators, nearly 50% used them for planning rather than implementation, suggesting that new patient navigator programs may be started in PY2. Evidence indicates that patient navigation increases CRC screening (26–28).

In the first program year, the overall screening rate increased by 4.4 percentage points. The CRCCP’s PY1 overall screening rate of 47.3% is much lower than the commonly cited 67.3% from the 2016 BRFSS. These results again confirm that grantees are working with clinics serving the intended populations and also indicate the significant gap in CRC screening rates between those reached by the CRCCP and the US population overall. Among FQHCs/CHCs participating in the CRCCP, the screening rate increased by 5.4 percentage points in PY1, compared with 1.6 percentage points for FQHCs nationally during 2015–2016 (https://bphc.hrsa.gov/uds/datacenter.aspx?year=2015). Given that PY1 included several or more months dedicated to program start-up (eg, grantees putting contracts in place, hiring staff), the time for EBI/SA implementation was limited. Consequently, we may observe more substantial increases in screening rates going forward as interventions are in place for a longer period. At the same time, given that 52.5% of clinics primarily used FIT/FOBT tests, there is a challenge of ensuring annual rescreening to maintain current levels.

The screening rate changes observed during the CRCCP PY1 varied by clinic characteristics and other process implementation factors. For instance, clinics with champions and screening policies had higher screening rate increases than those without a champion or policy. Many public health studies have established that champions contribute to improved outcomes (29). Screening policies may be associated with more organized screening approaches in which higher screening rates are likely. Of note, clinics with 3 or more EBIs in place at the end of PY1 had higher screening rate increases than clinics with fewer EBIs, suggesting a possible dose effect. This is similar to what the Community Guide has reported (25). Longitudinal data will allow CDC to examine trends and better assess factors associated with screening rate changes.

The evaluation of federally funded programs in multiple US states is challenging, given the complexity and diversity of programs and strategic implementation in the unique environment of individual states. CDC’s evaluation approach addresses these challenges by working closely with grantees to collect clinic-level process and outcome data. Involving stakeholders, developing strong data collection and reporting systems, and communicating frequently with grantees have helped CDC institute a strong evaluation and better understand contextual factors that affect the data interpretation. Most importantly, the evaluation design allows CDC to track implementation progress and outcomes in a more timely fashion and make programmatic adjustments as needed.

We noted some limitations of this PY1 evaluation. First, some interventions were in place for less than a year, given the time needed to start programs. Second, EHRs often needed improvements to produce accurate screening rates at the population level, leaving room for further improvements in the accuracy and reliability of screening rate measurement. Technical assistance provided to clinics played a crucial role in improving their capacity to report quality data. Third, given real-world program implementation, we cannot isolate the effects of factors, such as temporal trends in CRC screening, on clinic screening rates. However, future years of longitudinal data will help identify factors associated with screening rate changes. Finally, improvement of screening delivery was beyond the scope of this evaluation.

Other aspects of our evaluation are under way. CDC is completing qualitative case studies with a subset of grantees to learn more about implementation, including how EBIs/SAs are selected and prioritized. An economic study of program implementation with 11 of the CRCCP grantees is in progress. The study will provide valuable information about costs and return on investment of the chosen EBIs. Sustainability of public health activities is essential to achieving long-term health outcomes. Therefore, CDC is examining whether the CRCCP model leads to sustained process and outcomes after CRCCP resources end. In particular, we are assessing whether EBIs/SAs become institutionalized health systems changes within the partner clinics without having to rely on CRCCP resources. When intervention sustainability is achieved, grantees could redirect CRCCP resources to additional clinic sites, leading to expanded reach and impact of the program.

The CRCCP shows promise, as evidenced by PY1 results. Grantees have collaborated with more than 400 clinics, integrating public health interventions in primary care settings by implementing EBIs/SAs and increasing CRC screening rates. The frequency of implementation support provided to clinics, screening rate monitoring, and screening rate validation suggest substantial engagement between grantees and clinics and may reflect a high intensity of CRCCP process implementation contributing to outcomes. We anticipate increasing reach over time as EBIs are sustained, allowing program resources to be shifted to additional clinics. Rural clinics, where screening rates were especially low, are an area for expansion. Early evaluation results suggest that several factors may support greater screening rate increases including implementing multiple EBIs, making free FOBT/FIT kits available, engaging a clinic champion, and having a CRC screening policy in place. CDC’s support may also improve EHR data capture to achieve more accurate measurement of screening outcomes. Integrating evidence-based public health activities in primary care settings can help achieve needed increases in CRC screening among underserved populations.
